# Gastric dysmotility in Parkinson's disease is not caused by alterations of the gastric pacemaker cells

**DOI:** 10.1038/s41531-019-0087-3

**Published:** 2019-07-26

**Authors:** Konstantin G. Heimrich, Veit Y. P. Jacob, Denise Schaller, Andreas Stallmach, Otto W. Witte, Tino Prell

**Affiliations:** 1 Department of Gastroenterology, Jena University Hospital, Jena, Germany; 2 Department of Neurology, Jena University Hospital, Jena, Germany; 3 Center for Healthy Ageing, Jena University Hospital, Jena, Germany

**Keywords:** Physiology, Parkinson's disease

## Abstract

The enteric nervous system is involved in the pathology of Parkinson´s disease and patients frequently have symptoms related to delayed gastric emptying. However, the pathophysiology of gastric dysmotility is yet not well understood. The objective of this study was to assess interdigestive gastric motility in Parkinson´s disease. Using an electromagnetic capsule system, the dominant gastric contraction frequency (primary outcome measure) and the gastric transit time were assessed in 16 patients with Parkinson´s disease and 15 young healthy controls after a fasting period of 8 h. Motor and non-motor symptoms were assessed using the Movement Disorder Society Unified Parkinson´s Disease Rating Scale III (MDS-UPDRS III), the Non-Motor Symptoms Questionnaire (NMS-Quest), and Hoehn & Yahr staging. The Gastroparesis Cardinal Symptom Index was used to record symptoms related to delayed gastric emptying. In healthy controls and patients with Parkinson's disease, the dominant contraction frequency was 3.0 cpm indicating normal function of interstitial cells of Cajal. In patients with Parkinson's disease, the gastric transit time was longer than in younger controls (56 vs. 21 min). The dominant contraction frequency and gastric transit time did not correlate with age, disease duration, Hoehn & Yahr stage, levodopa equivalent daily dose, MDS-UPDRS III, NMS-Quest, and Gastroparesis Cardinal Symptom Index. Changes of gastric motility in Parkinson´s disease are not caused by functional deficits of the gastric pacemaker cells, the interstitial cells of Cajal. Therefore, gastroparesis in Parkinson's disease can be attributed to disturbances in neurohumoral signals via the vagus nerve and myenteric plexus.

## Introduction

Parkinson's disease (PD) is a multisystem neurodegenerative disorder characterized by motor and non-motor symptoms.^1^ The involvement of the entire gastrointestinal tract can cause a plethora of gastrointestinal symptoms.^2^ These symptoms can originate from functional and structural changes in the gut and its associated neural structures.^3,4^ It was hypothesized that pathological processes could be initiated in the enteric nervous system and spread to the central nervous system via the vagus nerve.^5^ However, this gut-to-brain scenario is still widely debated,^6^ and there is an urgent need to better characterize the enteric nervous system pathology in PD.^7^ Moreover, the assessment of gastric motility and gastric emptying is important for the diagnosis of gastroparesis, which contributes to motor fluctuations.

**Fig. 1:**
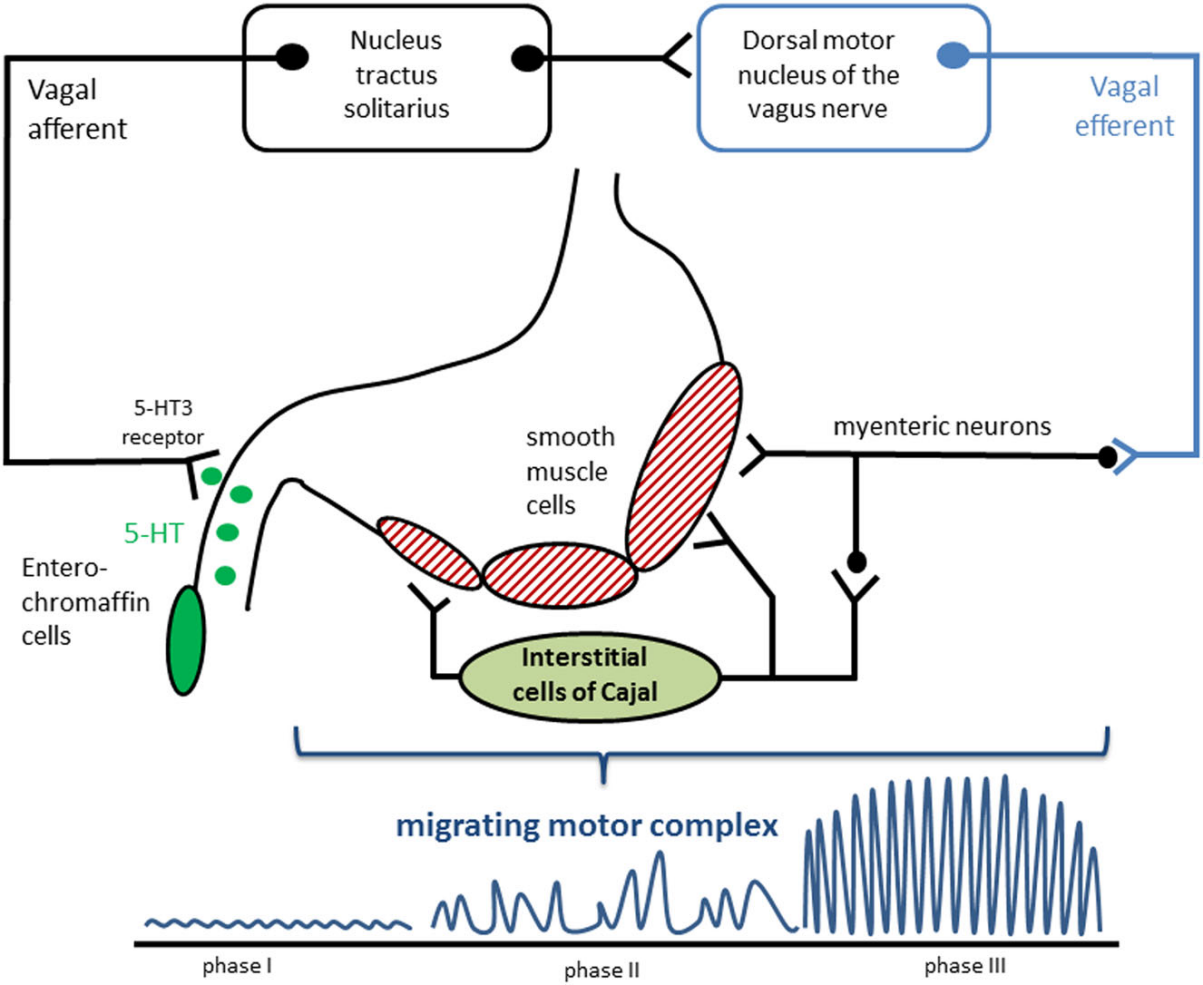
Physiology of gastric motility and the gastric migrating motor complex. Motilin and increased luminal pressure stimulate the release of 5-HT from duodenal enterochromaffin cells. The released 5-HT activates 5-HT3 receptors, and the signal is transduced via the vagus nerve afferents to the nucleus tractus solitarius. The vagus nerve stimulates gastric smooth muscle cells via myenteric neurons and interstitial cells of Cajal. Thus, motilin and 5-HT induce phase II and III of the migrating motor complex via the vago-vagal reflex. Figure adapted from Takahashi and Itoh.^11,12^

To understand functional disturbances of gastral motility in PD, one has to recapitulate the physiology of a coordinated gastric secretion and motility. In general, gastric motility can be subdivided into digestive (after food intake) and interdigestive (fasting state) motility. The interdigestive motility, also referred to as migrating motor complex, is a cyclic pattern of electromechanical activity in the gastrointestinal smooth muscle cells that can be partitioned into three phases by means of different contraction patterns (Fig. 1). Phase I is a period of smooth muscle quiescence lasting up to 60 min. In phase I the interstitial cells of Cajal generate gastric slow waves that occur with a frequency of 3 counts per minute (cpm). These spontaneous depolarizations and repolarizations cannot induce full muscle contractions. Additional neurohumoral signals via the vagus nerve and myenteric plexus are needed to increase the membrane potential above this threshold and to induce spike-wave activity, which is then associated with muscle contraction.^8^ In phase II intermittent contractions of varying strength and duration occur and progressively increase in frequency. Approximately every second slow wave is associated with the occurrence of corresponding spike potentials. In phase III the motor activity is strongest, and almost every slow wave is accompanied by a spike. Therefore, the ratio between slow waves and contractions is 1:1 with the maximum contraction frequency of 3 cpm. Peristaltic contractions occur. Phase III ensures the elimination of indigestible, larger food particles between the meals (“housekeeper function“) because the pylorus remains open during this phase III in contrast to digestive motility.

To date, measuring gastric motility has been restricted by several limitations inherent in the available measurement techniques (i.e. electrogastrography, ^13^C-acetate breath test, and scintigraphy). Gastric scintigraphy directly assesses mechanical gastric emptying, but patients are exposed to radiation and the method cannot provide information about gastric contractility. The ^13^C-acetate breath test is an indirect measure of gastric emptying, but mechanical gastric emptying, small intestine absorption, and liver metabolism can bias the results.^9^ The SmartPill™ consists of an indigestible capsule which measures pressure, pH, temperature, and indirectly a complete transit profile during passing the whole gastrointestinal tract. However, it cannot assess the orientation of the capsule and the contractile activity. Magnetic tracking systems are novel methods to overcome these limitations by assessing gastrointestinal motility based on the localization and orientation of small wireless magnetic markers inside the human body. Therefore, we studied gastric motility patterns in PD by using a high-resolution three-dimensional (3D) magnetic monitoring system. We aimed to gain new insight into functional aspects of gastric motility in PD. We focused on the physiology of gastric motility patterns related to function of the interstitial cells of Cajal.

## Results

### Clinical and demographical characteristics

Sixteen patients with PD with moderate motor impairment and a mean of 11 non-motor symptoms according to the NMS-Quest were studied (detailed in Table 1). All patients received levodopa (with or without entacapone or opicapone), in addition two patients received rotigotine, two patients pramipexole, and two patients safinamide.

**Table 1:** Clinical characteristics of patients with Parkinson´s disease

Sex (female, *n*, %)	6 (37.5)
Age (mean, SD, years)	74.8 (7.1)
Hoehn & Yahr (median, IQR)	3 (1)
Disease duration (mean, SD, years)	11.2 (8)
Levodopa equivalent daily dose (mean, SD, mg)	930 (480)
MDS-UPDRS III (mean, SD)	37 (20)
MDS-UPDRS total (mean, SD)	83 (43)
Non-motor symptoms questionnaire total (mean, SD)	11 (5)
Gastric transit time (mean, SD, min)	56 (62)
Gastric contractions (mean, SD, cpm)	3.0 (0.5)
Proton pump inhibitors (*n*, %)	6 (37.5)

### Frequency of gastric motility

**Fig. 2:**
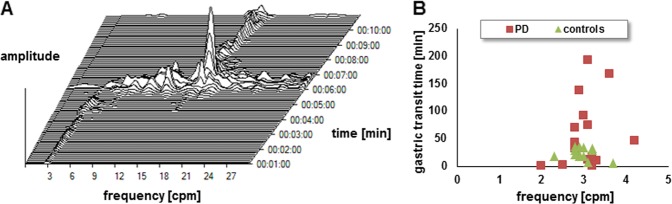
Phases of the migrating motor complex. **a** Fast Fourier transformation into a pseudo-three-dimensional running spectrum graph over a period of 10 min in a representative example of 3D-MAGMA recordings. The period in which the frequency is 3 cpm corresponds to phase I of the migrating motor complex. Then, a phase of intermittent contractions of varying strength and duration occur (phase II). In phase III, the motor activity is strongest and the capsule is leaving the stomach. Finally, the capsule reaches the duodenum with a frequency of approx. 15 cpm. **b** Distribution of gastric transit time and the dominant frequency in patients with Parkinson´s disease and young healthy controls

A representative example of 3D-MAGMA recordings using the FFT algorithm into a pseudo-3D running spectrum graph is shown in Fig. 2a. In the young healthy controls, we recorded periodical movements of the magnetic capsule at a frequency of 3.0 ± 0.3 cpm [95% CI 2.8–3.1] as long as the marker was in the stomach. Also, in patients with PD, the dominant frequency was 3.0 ± 0.5 cpm [95% CI 2.8–3.3] (*p* = 0.66) (Fig. 2b). The frequency of movements per minute did not correlate with age, disease duration, Hoehn & Yahr stage, levodopa equivalent daily dose, motor (MDS-UPDRS III) or non-motor burden (NMS-Quest), and did not significantly differ between PD patients taking proton pump inhibitors and PD patients who did not take proton pump inhibitors.

### Gastric transit time

In patients with PD, the mean GCSI was 1.0, indicating mild gastric symptom severity. Most patients reported post-prandial fullness (*n* = 8, 50%) and early satiety (*n* = 9, 56%). In patients with PD, the mean gastric transit time of the marker was 56 ± 62 min. The gastric transit time was highly heterogeneously distributed and ranged from 1 to 192 min. The gastric transit time in patients with PD was longer than in the young healthy control group (21 ± 11 min, range 2–36 min). The gastric transit time did not significantly correlate with the GCSI. Neither gastric transit time nor GCSI was correlated with age, disease duration, Hoehn & Yahr stage, levodopa equivalent daily dose, MDS-UPDRS III, or NMS-Quest. Gastric transit time did not differ between PD patients taking proton pump inhibitors and PD patients who did not take proton pump inhibitors (14 ± 54 vs. 45 ± 67 min).

### Methodological aspects during measurements

The duration of assessment ranged from 23 to 192 min. Four patients experienced wearing-off with slightly increasing rigor and bradykinesia during the measurement. None of the patients developed dyskinesias or relevant tremor which might interfere with the measurement. Two patients with camptocormia had problems to sit in the chair for the whole assessment. In four subjects, the capsule left the stomach within 3 min, which influences the validity of mean gastric transit time and made an evaluation of the dominant frequency more difficult.

## Discussion

The 3D-MAGMA system is able to determine the gastric transit time and gastric motility patterns in patients with PD. In our study we focused on the interdigestive motility period where the motility pattern can be described by the migrating motor complex (Fig. 1). In the first phase of the migrating motor complex, the interstitial cells of Cajal generate gastric slow waves with an intrinsic frequency of 3 cpm. The gastric peristalsis heavily depends on a proximal-to-distal gradient in this slow-wave frequency. It was hypothesized that two different patterns of contraction of gastric smooth muscle cells may exist: minor contractions facilitated by slow-wave activity and major contractions caused by spike potentials superimposed over slow-wave potentials. These spike potentials are evoked by additional neurohumeral stimulation and trigger phasic gastric contractions.^10^ As demonstrated before and replicated in our healthy young control group, every gastric slow wave is physiologically associated with a gastric tonic muscle contraction.^8^ Our study also shows that in PD, every gastric slow wave goes along with a gastric contraction, indicating that the existing slow-wave-generating interstitial cells of Cajal are functional in PD. Therefore, PD-related gastroparesis is not caused by a damage of the electrical pacemaking function of the cells of Cajal. The additional neurohumoral stimulation that is needed for major contractions is mediated via the vagus nerve and endogenous 5-HT (Fig. 1).^11,12^ In response to luminal pressure increase and motilin, 5-HT is released from the enterochromaffin cells into the lumen and here it stimulates vagal sensory fibers. Through the nucleus tractus solitarius and dorsal motor nucleus of the vagus, the information of increased luminal pressure is transferred and the vagal efferent stimulates the release of acetylcholine from the myenteric plexus, resulting in muscle contractions.^11^ Therefore, alterations in gastric motility in PD can be explained by alterations of the vagus nerve and the enteric nervous system. This is in line with the accumulation of α-synuclein aggregates in the vagal nerve as well as in myenteric neurons, where it leads to a disrupted innervation of intestinal smooth muscle cells.^2,13^ If the migrating motor complex is impaired, the gastric emptying is delayed, and this has been associated with small intestinal bacterial overgrowth, chronic inflammation, pain, and constipation.^11^

The 3D-MAGMA system also allows the determination of a gastric transit time. However, we have some concerns about using the method for measuring transit time in PD. The main problem in our opinion is the high interindividual variability of gastric transit time measures. Therefore large age-matched sample sizes are necessary, which is, however, difficult to achieve due to PD-related problems (i.e. long measure time, problems to sit, fluctuations etc.). Nevertheless, our results in terms of gastric transit time have to be discussed in the following. Using a similar system, the ambulatory 3D-Transit System (Motilis Medica SA, Lausanne, Switzerland), Knudsen et al.^14^ recently found no significant delay of gastric transit time in patients with PD compared to controls. We observed a longer gastric transit time in PD compared with that in young healthy controls. This is probably mainly caused by three outliers. Theoretically, this could also be explained by an age-related decrease in gastric motility. However, the age-related changes in gastric motility are poorly understood. It seems to be more likely that motor function of the stomach is relatively well preserved with healthy aging, with very modest slowing of gastric emptying.^15^ Nevertheless, some studies support an age-related selective decrease in the number of cholinergic neurons in the enteric nervous system, but also showed a progressive loss of interstitial cells of Cajal in the stomach and colon throughout adult life.^16,17^ In line with our results, these changes appear to have surprisingly little effect on gastrointestinal motor function. However, the measured mean gastric transit time also was shorter in our cohort than in the study by Knudson et al. There are several reasons for this difference. First, we examined interdigestive motility and Knudsen et al. assessed digestive gastric motility. We chose a stationary setting in which the subjects were requested to not move or eat for several hours or until the measurement was finished. In contrast, Knudsen et al. used an ambulatory setting and measurements started after ingestion of a standardized meal. In addition, there are technical differences. The 3D-MAGMA and the Motilis 3D-Transit system are both ingestible capsule systems that work on an electromagnetic basis. However, 3D-MAGMA has a smaller capsule size of 18 × 6 mm (vs. 21 × 8 mm), more sensors (27 vs. 4), and a measurement frequency of up to 50 Hz (vs. 5–10 Hz). Taken together, we can confirm the applicability of 3D systems in assessing gastric motility in PD. However, the results differ depending on the different motility periods and the studied cohort.

There are conflicting results in terms of the association between clinical variables and gastric motility measures. Using the ^13^C-acetate breath test, Tanaka et al.^18,19^ demonstrated that gastric emptying time is neither influenced by motor fluctuation nor different between untreated and treated PD patients. Whereas Hardoff et al.^20^ did not find any difference between early and later stage PD patients using scintigraphy, Goetze et al.^21^ showed a significant increase of gastric emptying time in later disease stages using the breath test. However, the gastric emptying time derived from scintigraphy or the breath test is not comparable to the gastric transit time measured with the magnetic system. Gastric transit time is determined as the time until the capsule leaves the stomach. In contrast, gastric emptying time describes the time until the retention of a radiolabeled meal reaches a defined percentage. Usually, enhanced gastric emptying time is defined as retention greater than 10% after 4 h.^22^ In contrast to gastric emptying time measures, there exists no cut-off for delayed emptying based on the gastric transit time. In our study, in 50% of PD patients gastric transit time was longer than the longest value in the healthy controls. Additionally, the gastric transit time showed a high variation and ranged from 1 to 192 min. We assume that the rapid pass-through in four of our patients is related to the phase of the migrating motor complex in which the capsule was administered. If the capsule is administered close to phase III, the motor activity is very strong and the capsule should leave the stomach quickly.

We did not observe correlations between gastric transit time and disease stage (Hoehn & Yahr), motor severity (MDS-UPDRS III), and non-motor burden (NMS-Quest). However, our study was not intended to study these associations and therefore these results have to be taken with caution. The lacking correlation with the Hoehn & Yahr stage is probably because the majority of patients (*n* = 9, 56%) were in Hoehn & Yahr stage 3. Of note, gastric transit time did not correlate with the GCSI. Therefore, this symptom questionnaire is probably not predictive and sensitive to determine a decrease of gastric dynamics in PD. And, conversely, a delayed gastric transit time can of course not explain multifactorial symptoms such as early satiety, bloating, nausea, and vomiting. Once again, it must be mentioned that the variation of gastric transit time in PD is high. Therefore a combination of electrogastrography and migrating motor complex-controlled administration of the capsule at the beginning of phase I would be an approach to explore if gastric transit time correlates with disease severity.

Our study has some limitations. We did not use an age-matched control group. Theoretically it is possible that one of the factors, like aging, decreases the frequency, while the other, like PD, increases the frequency with the two influences cancelling each other out. However, we think this is highly unlikely. In another study using the same system in older healthy subjects (mean age 40.4 years) the frequency was similar to our study. Nevertheless, we cannot fully exclude that aging processes might influence the frequency and therefore reference values for age-matched healthy control groups are necessary. We recruited patients from the neurological wards and preferably patients with advanced disease stage. We carefully selected patients without any confounding comorbidity, but this of course limits the generalizability of our results. On the other hand we aimed to avoid bias through comorbidities affecting gastric motility. There are no age-related reference values for 3D-MAGMA. However, these are necessary to fully understand age-related and PD-related changes of gastral dynamics. Our study was conducted in the medication on-phase. Therefore changes in gastric transit time could also be related to PD medication. This has to be addressed in further studies. Although one study showed that administration of omeprazole did not affect gastric motility,^23^ we cannot fully rule out that proton pump inhibitors can influence gastric emptying and gastric symptoms.

3D-MAGMA is a highly sensitive motility measurement tool offering new insights into the pathophysiology of gastric motility in PD. In addition to the assessment of mechanical gastric emptying, the contraction frequency allows studying functional aspects of gastric emptying. Changes of gastric motility in PD are not caused by functional deficits of the interstitial cells of Cajal. Therefore, gastroparesis in PD is probably related to disturbances in neurohumoral signals via the vagus nerve and myenteric plexus. Further studies in age-matched healthy controls are necessary to determine age-specific reference values.

## Methods

### Subjects and clinical assessment

This cross-sectional study was approved by the local Ethics Committee of the Jena University Hospital (No. 4572-10/15) and has been performed in compliance with the Declaration of Helsinki. All subjects gave written informed consent. Sixteen patients with PD with a mean age of 74.8 ± 7.1 [95% confidence interval (CI) 71.7–79.4] years participated in the study. To rule out ageing-related changes and to compare the motility patterns of PD patients with the physiological state, sex-matched healthy young students from the University of Jena were recruited as controls (mean age 27 ± 2.5 years; *p* < 0.001, [95% CI 25.1–27.9]). Patients with PD were recruited from the wards of the Department of Neurology, Jena University Hospital. The inclusion criteria were as follows: PD diagnosis according to Movement Disorder Society (MDS) diagnosis criteria, stable under dopaminergic treatment, and able to sit up to 3 h in order to avoid artefacts due to body movements. The exclusion criteria were as follows: agitation, PD dementia (Montreal Cognitive Assessment <21 points), neuropathy, diabetes mellitus, gastroesophageal reflux, liver disease, deep brain stimulation, levodopa/carbidopa enteral infusion, apomorphine infusion, anticholinergic agents, opiates, radiation, or abdominal surgery. All tests were conducted during the medication ON phase. The MDS-sponsored revision of the Unified Parkinson's Disease Rating Scale (MDS-UPDRS),^24^ the revised Non-Motor Symptoms Questionnaire (NMS-Quest), and Hoehn & Yahr staging were used to evaluate motor and non-motor symptoms. The Gastroparesis Cardinal Symptom Index (GCSI) was used to assess symptoms related to gastroparesis (contact information and permission to use: Mapi Research Trust, Lyon, France—https://eprovide.mapi-trust.org).^25,26^ The GCSI consists of three subscales: nausea/vomiting (3 items), post-prandial fullness/early satiety (4 items), and bloating (2 items). The GCSI total score ranges from 0 to 5, and higher values indicate greater symptom severity.

### 3D-MAGMA

**Fig. 3:**
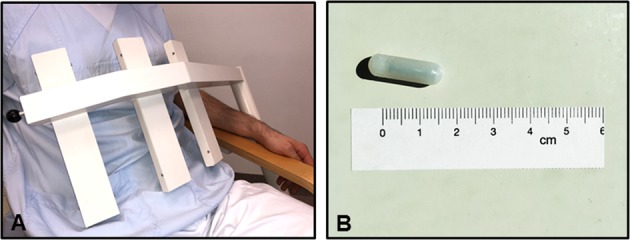
3D-MAGMA system. **a** 3D-MAGMA sensor arrangement. **b** 3D-MAGMA capsule

The magnetic monitoring system 3D-MAGMA (Innovent, Jena, Germany) was used to assess gastric motility. It contains 27 highly sensitive magnetic field sensors that are arranged contact free above the abdomen (Fig. 3a). The system measures the magnetic field of a small permanent magnetic capsule (neodymium–iron–boron covered with indigestible polyethylene; outer diameter 6 mm, overall length 18 mm) (Fig. 3b) and calculates its 3D position and orientation. The capsule emits an electromagnetic field that is monitored by the detector with a measurement frequency of up to 50 Hz. The capsule is carried by natural peristalsis through the complete gastrointestinal tract. In the unlikely case that the capsule gets stuck in the gastrointestinal tract, the possibility of endoscopic removal was maintained. Using nonlinear optimization algorithms 3D-MAGMA allows a precise localization of the capsule as well as the contractile activity of the examined part of the gastrointestinal tract.^27^ As endoscopically shown, the capsule is positioned close to the inner stomach wall after ingestion.^8^ The accuracy of the data relies on the available magnetic field strength from the capsule and therefore depends on the distance between detector and capsule. Within the whole gastrointestinal tract, the position error is less than 5 mm. The field strength has a range of 10^−8^ to 10^−5^ T. In contrast, muscle movement of the stomach or intestinal wall induces a magnetic field of only 10^−12^ T,^28^ which cannot be detected by 3D-MAGMA. The dominant frequency can be calculated using the fast Fourier transform (FFT) algorithm. As demonstrated previously, the intra- and interindividual variability of the measurements at different days showed very good, high reproducibility.^8^

The measurements always started in the morning at 8 a.m. after taking the morning medication. Measurement in patients with PD started in the medication ON phase. Participants were comfortably seated in a diagnostic armchair, and the magnetic field sensor system was adjusted. The subjects had been fasting for at least 8 h before the examination. The capsule was orally administered with 70 mL of water, which does not affect the motility status.^29^ The stomach was in the cycle of interdigestive motility after taking the marker. The patients were asked to stay in the chair for up to 4 h or until the capsule passes the stomach.

### Statistical analyses

The SPSS statistical computer package (version 25.0; IBM Corporation, Armonk, NY, USA) was used for all statistical analyses. The primary outcome measure was the dominant gastric frequency, because this is related to function of the interstitial cells of Cajal. The gastric transit time was not an outcome measure, because ageing processes might influence gastric emptying and for this purpose an aged-matched control group would be necessary. Therefore, no confirmatory tests were performed for group comparison of gastric transit time. Given the mean frequency and standard deviation in the healthy control group the sample size calculation (two-sided equality, power 0.8, alpha 5%, assumed group difference 0.5 cpm) indicated that 11 subjects were necessary for each group. Prior to statistical analysis, data were checked for outliers and for normality using the Shapiro–Wilk test. Descriptive analyses were used to describe clinical characteristics. Correlation between gastric transit time and clinical variables was tested using the Pearson correlation for normal distribution and Spearman correlation for non-normally distributed data. Group comparisons were performed using the *t-*test for normal distribution and the Mann–Whitney *U* test for non-normally distributed variables (two-sided). The significance level was set at *p* < 0.05.

### Reporting Summary

Further information on research design is available in the Nature Research Reporting Summary linked to this article.

## Supplementary information

Reporting Summary

## Data Availability

Data are available for scientific purpose from the corresponding author upon reasonable request.

## Supplementary information

**Supplementary information** accompanies the paper on the *npj Parkinson’s Disease* website (10.1038/s41531-019-0087-3).
